# 1-(2,4-Difluoro­phen­yl)thio­urea

**DOI:** 10.1107/S1600536812031625

**Published:** 2012-07-18

**Authors:** Hoong-Kun Fun, Ching Kheng Quah, Prakash S. Nayak, B. Narayana, B. K. Sarojini

**Affiliations:** a X-ray Crystallography Unit, School of Physics, Universiti Sains Malaysia, 11800 USM, Penang, Malaysia; b Department of Studies in Chemistry, Mangalore University, Mangalagangotri 574 199, India; c Department of Chemistry, P. A. College of Engineering, Nadupadavu, Mangalore 574 153, India

## Abstract

The asymmetric unit of the title compound, C_7_H_6_F_2_N_2_S, consists of two independent mol­ecules, with comparable geometries. In one mol­ecule, the thio­urea moiety is essentially planar (r.m.s. deviation = 0.014 Å) and it forms a dihedral angle of 78.67 (9)° with the benzene ring. The corresponding r.m.s. deviation and dihedral angle for the other mol­ecule are 0.011 Å and 81.71 (8)°, respectively. In both mol­ecules, one of the F atoms is disordered over two positions with refined site occupancies of 0.572 (3):0.428 (3) and 0.909 (2):0.091 (2), respectively. In the crystal, mol­ecules are linked *via* N—H⋯S and C—H⋯F hydrogen bonds into two-dimensional networks parallel to (010).

## Related literature

For general background to and the related structures of the title compound, see: Fun *et al.* (2012*a* ▶,*b* ▶); Sarojini *et al.* (2007 ▶). For standard bond-length data, see: Allen *et al.* (1987 ▶). For the stability of the temperature controller used for the data collection, see: Cosier & Glazer (1986 ▶).
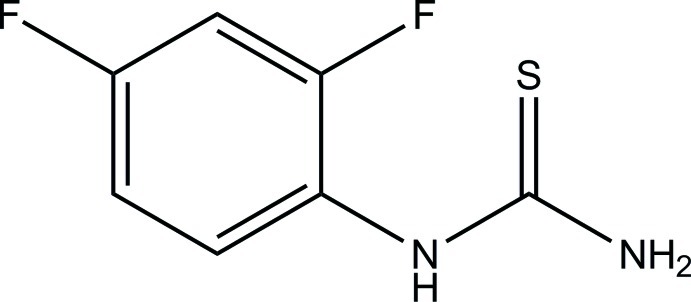


## Experimental

### 

#### Crystal data

C_7_H_6_F_2_N_2_S
*M* *_r_* = 188.20
Monoclinic, 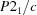

*a* = 6.4260 (7) Å
*b* = 36.908 (4) Å
*c* = 6.6821 (7) Å
β = 100.464 (2)°
*V* = 1558.4 (3) Å^3^
*Z* = 8
Mo *K*α radiation
μ = 0.39 mm^−1^
*T* = 100 K
0.36 × 0.14 × 0.09 mm

#### Data collection

Bruker SMART APEXII DUO CCD area-detector diffractometer
Absorption correction: multi-scan (*SADABS*; Bruker, 2009 ▶) *T*_min_ = 0.874, *T*_max_ = 0.967
13654 measured reflections
3553 independent reflections
3082 reflections with *I* > 2σ(*I*)
*R*_int_ = 0.031

#### Refinement

*R*[*F* ^2^ > 2σ(*F* ^2^)] = 0.033
*wR*(*F* ^2^) = 0.077
*S* = 1.06
3553 reflections
255 parameters
4 restraints
H atoms treated by a mixture of independent and constrained refinement
Δρ_max_ = 0.55 e Å^−3^
Δρ_min_ = −0.36 e Å^−3^

### 

Data collection: *APEX2* (Bruker, 2009 ▶); cell refinement: *SAINT* (Bruker, 2009 ▶); data reduction: *SAINT*; program(s) used to solve structure: *SHELXTL* (Sheldrick, 2008 ▶); program(s) used to refine structure: *SHELXTL*; molecular graphics: *SHELXTL*; software used to prepare material for publication: *SHELXTL* and *PLATON* (Spek, 2009 ▶).

## Supplementary Material

Crystal structure: contains datablock(s) global, I. DOI: 10.1107/S1600536812031625/kj2208sup1.cif

Structure factors: contains datablock(s) I. DOI: 10.1107/S1600536812031625/kj2208Isup2.hkl

Supplementary material file. DOI: 10.1107/S1600536812031625/kj2208Isup3.cml

Additional supplementary materials:  crystallographic information; 3D view; checkCIF report

## Figures and Tables

**Table 1:** Hydrogen-bond geometry (Å, °)

*D*—H⋯*A*	*D*—H	H⋯*A*	*D*⋯*A*	*D*—H⋯*A*
N1*A*—H1N*A*⋯S1*B*	0.794 (19)	2.586 (19)	3.3485 (15)	161.7 (19)
N2*A*—H2N*A*⋯S1*B*	0.81 (2)	2.77 (3)	3.499 (2)	151 (2)
N2*A*—H3N*A*⋯S1*B* ^i^	0.85 (2)	2.65 (2)	3.504 (2)	175.2 (16)
N1*B*—H1N*B*⋯S1*A* ^ii^	0.88 (2)	2.49 (2)	3.3273 (15)	158.9 (17)
N2*B*—H2N*B*⋯S1*A* ^ii^	0.88 (2)	2.76 (2)	3.5179 (19)	146.4 (18)
N2*B*—H3N*B*⋯S1*A* ^iii^	0.82 (3)	2.66 (3)	3.4592 (19)	167 (2)
C4*B*—H4*BA*⋯F1*B* ^i^	0.95	2.50	3.094 (2)	121
C5*B*—H5*BA*⋯F1*B* ^i^	0.95	2.52	3.111 (2)	121

Symmetry codes: (i) 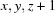; (ii) 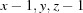; (iii) 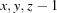.

**Fig. 1.**
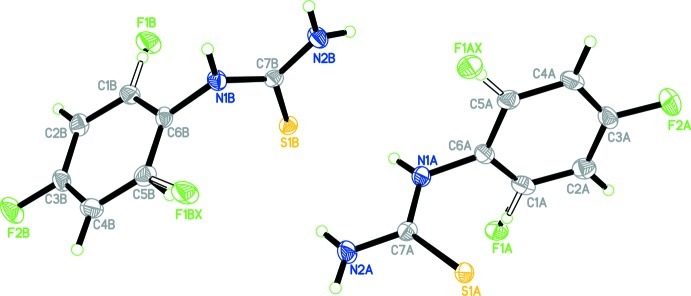
The asymmetric unit of the title compound showing 50% probability displacement ellipsoids for non-H atoms. Both major and minor disorder component are shown.

**Fig. 2.**
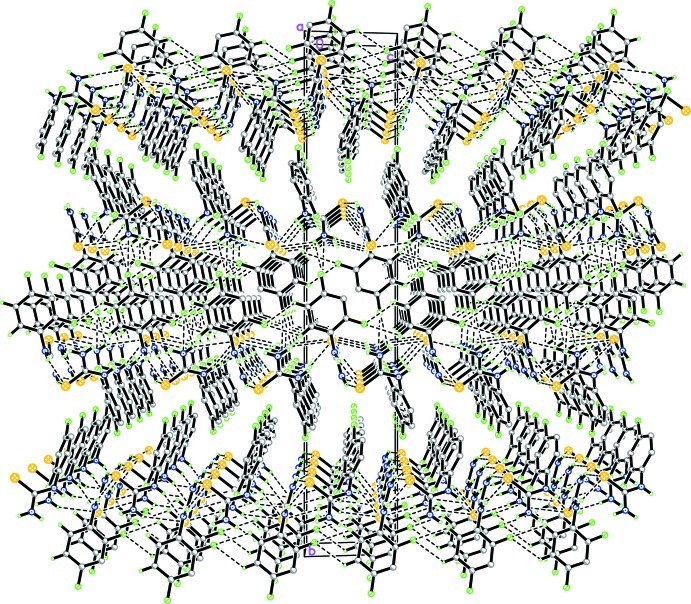
The crystal structure of the title compound, viewed along the a axis. H atoms not involved in hydrogen bonds (dashed lines) have been omitted for clarity. Only major component of disorder is shown.

## supplementary crystallographic information

### Comment

In continuation of our work on the synthesis of thiourea derivatives (Fun *et al.*, 2012*a*, 2012*b*; Sarojini *et al.*, 2007), the title compound is prepared and its crystal structure is reported here.

The asymmetric unit (Fig. 1) of the title compound consists of two independent molecules (*A* and *B*), with comparable geometries. In molecule *A*, thiourea moiety (S1A/N1A/N2A/C7A) is essentially planar (r.m.s. deviation = 0.014 Å) and it forms a dihedral angle of 78.67 (9)° with the benzene ring (C1A-C6A). The corresponding r.m.s. deviation and dihedral angle for molecule *B* are 0.011 Å and 81.71 (8)°, respectively. Bond lengths (Allen *et al.*, 1987) and angles are within normal ranges and are comparable to related structures (Fun *et al.*, 2012*a*, 2012*b*). The fluorine atoms (F1A/F1B) of both molecules are disordered over two positions with refined site-occupancies of 0.572 (3):0.428 (3) and 0.909 (2): 0.091 (2), respectively.

In the crystal structure, Fig. 2, molecules are linked *via* intermolecular N—H···S and C—H···F hydrogen bonds (Table 1) into two-dimensional networks parallel to (010).

### Experimental

2,4-Difluoroaniline (0.84 mL, 0.0081 mol) was refluxed with potassium thiocyanate (1.4 g, 0.0142 mol) in 20 mL of water and 1.6 mL of concentrated HCl for 3 h. The reaction mixture was then cooled to room temperature and stirred overnight. The precipitated product was then filetred, washed with water, dried and recrystallised from acetone and toluene (1:1) mixture by slow evaporation method (*m.p.* 441-443K).

### Refinement

N-bound hydrogen atoms were located in a difference Fourier map and refined freely with N–H = 0.79 (2)–0.88 (2) Å. The remaining H atoms were positioned geometrically and refined using a riding model with C–H = 0.95 Å and *U*_iso_(H) = 1.2 *U*_eq_(C). The fluorine atoms (F1A/F1B) of both molecules are disordered over two positions with refined site-occupancies of 0.572 (3):0.428 (3) and 0.909 (2): 0.091 (2), respectively. The same U_ij_ parameters were used for atom pair F1B/F1BX.

### Crystal data

**Table:**

C_7_H_6_F_2_N_2_S	*F*(000) = 768
*M**_r_* = 188.20	*D*_x_ = 1.604 Mg m^−^^3^
Monoclinic, *P*2_1_/*c*	Mo *K*α radiation, λ = 0.71073 Å
Hall symbol: -P 2ybc	Cell parameters from 5197 reflections
*a* = 6.4260 (7) Å	θ = 3.2–32.0°
*b* = 36.908 (4) Å	µ = 0.39 mm^−^^1^
*c* = 6.6821 (7) Å	*T* = 100 K
β = 100.464 (2)°	Plate, colourless
*V* = 1558.4 (3) Å^3^	0.36 × 0.14 × 0.09 mm
*Z* = 8	

### Data collection

**Table:**

Bruker SMART APEXII DUO CCD area-detector diffractometer	3553 independent reflections
Radiation source: fine-focus sealed tube	3082 reflections with *I* > 2σ(*I*)
Graphite monochromator	*R*_int_ = 0.031
φ and ω scans	θ_max_ = 27.5°, θ_min_ = 1.1°
Absorption correction: multi-scan (*SADABS*; Bruker, 2009)	*h* = −8→8
*T*_min_ = 0.874, *T*_max_ = 0.967	*k* = −47→47
13654 measured reflections	*l* = −8→8

### Refinement

**Table:**

Refinement on *F*^2^	Primary atom site location: structure-invariant direct methods
Least-squares matrix: full	Secondary atom site location: difference Fourier map
*R*[*F*^2^ > 2σ(*F*^2^)] = 0.033	Hydrogen site location: inferred from neighbouring sites
*wR*(*F*^2^) = 0.077	H atoms treated by a mixture of independent and constrained refinement
*S* = 1.06	*w* = 1/[σ^2^(*F*_o_^2^) + (0.0271*P*)^2^ + 1.0603*P*] where *P* = (*F*_o_^2^ + 2*F*_c_^2^)/3
3553 reflections	(Δ/σ)_max_ = 0.001
255 parameters	Δρ_max_ = 0.55 e Å^−^^3^
4 restraints	Δρ_min_ = −0.36 e Å^−^^3^

### Special details

**Table:**

Experimental. The crystal was placed in the cold stream of an Oxford Cryosystems Cobra open-flow nitrogen cryostat (Cosier & Glazer, 1986) operating at 100.0 (1) K.
Geometry. All esds (except the esd in the dihedral angle between two l.s. planes) are estimated using the full covariance matrix. The cell esds are taken into account individually in the estimation of esds in distances, angles and torsion angles; correlations between esds in cell parameters are only used when they are defined by crystal symmetry. An approximate (isotropic) treatment of cell esds is used for estimating esds involving l.s. planes.
Refinement. Refinement of F^2^ against ALL reflections. The weighted R-factor wR and goodness of fit S are based on F^2^, conventional R-factors R are based on F, with F set to zero for negative F^2^. The threshold expression of F^2^ > 2sigma(F^2^) is used only for calculating R-factors(gt) etc. and is not relevant to the choice of reflections for refinement. R-factors based on F^2^ are statistically about twice as large as those based on F, and R- factors based on ALL data will be even larger.

### Fractional atomic coordinates and isotropic or equivalent isotropic displacement parameters (Å^2^)

**Table:**

	*x*	*y*	*z*	*U*_iso_*/*U*_eq_	Occ. (<1)
F1A	0.9955 (3)	0.14057 (5)	0.6462 (3)	0.0235 (5)	0.572 (3)
F1AX	0.4561 (4)	0.21131 (7)	0.3297 (4)	0.0259 (7)	0.428 (3)
F2A	1.13116 (19)	0.26104 (3)	0.46796 (18)	0.0356 (3)	
S1A	0.59786 (6)	0.167502 (10)	0.91274 (6)	0.01619 (10)	
N1A	0.5812 (2)	0.14793 (4)	0.5240 (2)	0.0209 (3)	
N2A	0.4287 (3)	0.10890 (4)	0.7173 (3)	0.0227 (3)	
C1A	0.9376 (3)	0.17296 (5)	0.5676 (3)	0.0223 (4)	
H1AA	0.9899	0.1505	0.6251	0.027*	0.428 (3)
C2A	1.0788 (3)	0.20077 (5)	0.5578 (3)	0.0240 (4)	
H2AA	1.2258	0.1980	0.6090	0.029*	
C3A	0.9968 (3)	0.23273 (5)	0.4704 (3)	0.0227 (4)	
C4A	0.7859 (3)	0.23758 (5)	0.3885 (3)	0.0221 (4)	
H4AA	0.7357	0.2597	0.3246	0.027*	
C5A	0.6497 (3)	0.20898 (5)	0.4030 (3)	0.0207 (4)	
H5AA	0.5035	0.2116	0.3475	0.025*	0.572 (3)
C6A	0.7219 (3)	0.17666 (4)	0.4967 (3)	0.0193 (3)	
C7A	0.5343 (2)	0.13960 (4)	0.7068 (3)	0.0167 (3)	
F1B	−0.1368 (2)	0.05450 (3)	−0.13223 (17)	0.0289 (3)	0.909 (2)
F1BX	−0.1259 (12)	0.0838 (3)	0.5203 (17)	0.0289 (3)	0.091 (2)
F2B	−0.33235 (18)	−0.03440 (3)	0.29943 (18)	0.0313 (3)	
S1B	0.32399 (6)	0.086866 (11)	0.19944 (6)	0.01690 (10)	
N1B	−0.0833 (2)	0.10552 (4)	0.1680 (2)	0.0178 (3)	
N2B	0.1372 (3)	0.15004 (4)	0.0948 (2)	0.0204 (3)	
C1B	−0.1715 (2)	0.04402 (5)	0.0510 (3)	0.0195 (3)	
H1BA	−0.1484	0.0513	−0.0796	0.023*	0.091 (2)
C2B	−0.2367 (2)	0.00890 (5)	0.0765 (3)	0.0207 (3)	
H2BA	−0.2598	−0.0080	−0.0327	0.025*	
C3B	−0.2663 (2)	−0.00024 (4)	0.2694 (3)	0.0201 (3)	
C4B	−0.2367 (3)	0.02342 (5)	0.4308 (3)	0.0226 (4)	
H4BA	−0.2606	0.0161	0.5610	0.027*	
C5B	−0.1706 (3)	0.05838 (5)	0.3979 (3)	0.0205 (3)	
H5BA	−0.1474	0.0752	0.5074	0.025*	0.909 (2)
C6B	−0.1382 (2)	0.06909 (4)	0.2074 (3)	0.0163 (3)	
C7B	0.1138 (3)	0.11572 (4)	0.1495 (2)	0.0157 (3)	
H1NA	0.538 (3)	0.1354 (5)	0.429 (3)	0.018 (5)*	
H2NA	0.390 (3)	0.0967 (6)	0.617 (4)	0.026 (6)*	
H3NA	0.396 (3)	0.1029 (5)	0.831 (3)	0.020 (5)*	
H1NB	−0.190 (3)	0.1202 (6)	0.124 (3)	0.024 (5)*	
H2NB	0.028 (3)	0.1640 (6)	0.052 (3)	0.026 (5)*	
H3NB	0.249 (4)	0.1570 (6)	0.067 (3)	0.025 (6)*	

### Atomic displacement parameters (Å^2^)

**Table:**

	*U*^11^	*U*^22^	*U*^33^	*U*^12^	*U*^13^	*U*^23^
F1A	0.0242 (9)	0.0180 (9)	0.0258 (10)	0.0068 (7)	−0.0019 (7)	0.0057 (7)
F1AX	0.0195 (12)	0.0277 (14)	0.0287 (14)	0.0035 (9)	0.0000 (10)	0.0036 (11)
F2A	0.0395 (6)	0.0311 (6)	0.0365 (7)	−0.0152 (5)	0.0080 (5)	0.0054 (5)
S1A	0.01840 (19)	0.01375 (19)	0.0162 (2)	−0.00093 (14)	0.00254 (15)	−0.00129 (15)
N1A	0.0306 (8)	0.0157 (7)	0.0157 (7)	−0.0058 (6)	0.0022 (6)	−0.0030 (6)
N2A	0.0322 (8)	0.0169 (7)	0.0195 (8)	−0.0079 (6)	0.0057 (7)	−0.0034 (6)
C1A	0.0331 (9)	0.0172 (8)	0.0179 (8)	0.0043 (7)	0.0081 (7)	0.0019 (7)
C2A	0.0246 (9)	0.0289 (9)	0.0195 (9)	0.0003 (7)	0.0072 (7)	0.0013 (7)
C3A	0.0323 (9)	0.0200 (8)	0.0180 (8)	−0.0069 (7)	0.0101 (7)	−0.0009 (7)
C4A	0.0350 (9)	0.0161 (8)	0.0162 (8)	0.0014 (7)	0.0070 (7)	0.0025 (7)
C5A	0.0277 (9)	0.0205 (8)	0.0136 (8)	0.0010 (7)	0.0025 (7)	−0.0015 (7)
C6A	0.0297 (9)	0.0151 (8)	0.0137 (8)	−0.0027 (6)	0.0060 (7)	−0.0024 (6)
C7A	0.0167 (7)	0.0144 (7)	0.0180 (8)	0.0028 (6)	0.0006 (6)	−0.0005 (6)
F1B	0.0454 (7)	0.0278 (6)	0.0145 (6)	−0.0080 (5)	0.0084 (5)	−0.0017 (5)
F1BX	0.0454 (7)	0.0278 (6)	0.0145 (6)	−0.0080 (5)	0.0084 (5)	−0.0017 (5)
F2B	0.0443 (7)	0.0172 (5)	0.0334 (6)	−0.0111 (5)	0.0098 (5)	0.0002 (5)
S1B	0.01641 (18)	0.01577 (19)	0.0178 (2)	0.00075 (14)	0.00125 (15)	−0.00053 (16)
N1B	0.0161 (7)	0.0138 (7)	0.0231 (7)	0.0012 (5)	0.0029 (6)	0.0021 (6)
N2B	0.0190 (7)	0.0161 (7)	0.0265 (8)	0.0003 (6)	0.0053 (6)	0.0039 (6)
C1B	0.0200 (8)	0.0221 (8)	0.0171 (8)	−0.0005 (6)	0.0050 (6)	0.0015 (7)
C2B	0.0230 (8)	0.0189 (8)	0.0205 (8)	−0.0031 (6)	0.0044 (7)	−0.0052 (7)
C3B	0.0203 (8)	0.0143 (8)	0.0255 (9)	−0.0026 (6)	0.0034 (7)	0.0014 (7)
C4B	0.0285 (9)	0.0215 (9)	0.0176 (8)	−0.0020 (7)	0.0034 (7)	0.0022 (7)
C5B	0.0236 (8)	0.0185 (8)	0.0181 (8)	0.0002 (6)	0.0003 (7)	−0.0028 (7)
C6B	0.0125 (7)	0.0137 (7)	0.0224 (9)	0.0004 (6)	0.0020 (6)	0.0002 (6)
C7B	0.0197 (8)	0.0166 (8)	0.0104 (7)	−0.0011 (6)	0.0020 (6)	−0.0022 (6)

### Geometric parameters (Å, º)

**Table:**

F1A—C1A	1.331 (2)	F1B—C1B	1.341 (2)
F1AX—C5A	1.254 (3)	F1BX—C5B	1.242 (12)
F2A—C3A	1.3572 (19)	F2B—C3B	1.3567 (19)
S1A—C7A	1.7079 (17)	S1B—C7B	1.7049 (16)
N1A—C7A	1.346 (2)	N1B—C7B	1.348 (2)
N1A—C6A	1.427 (2)	N1B—C6B	1.427 (2)
N1A—H1NA	0.79 (2)	N1B—H1NB	0.88 (2)
N2A—C7A	1.329 (2)	N2B—C7B	1.334 (2)
N2A—H2NA	0.81 (2)	N2B—H2NB	0.88 (2)
N2A—H3NA	0.85 (2)	N2B—H3NB	0.82 (2)
C1A—C2A	1.379 (3)	C1B—C2B	1.382 (2)
C1A—C6A	1.387 (3)	C1B—C6B	1.383 (2)
C1A—H1AA	0.9500	C1B—H1BA	0.9500
C2A—C3A	1.377 (3)	C2B—C3B	1.378 (3)
C2A—H2AA	0.9500	C2B—H2BA	0.9500
C3A—C4A	1.379 (3)	C3B—C4B	1.374 (2)
C4A—C5A	1.386 (2)	C4B—C5B	1.389 (2)
C4A—H4AA	0.9500	C4B—H4BA	0.9500
C5A—C6A	1.387 (2)	C5B—C6B	1.384 (2)
C5A—H5AA	0.9500	C5B—H5BA	0.9500
			
C7A—N1A—C6A	122.52 (15)	C7B—N1B—C6B	123.25 (14)
C7A—N1A—H1NA	119.4 (15)	C7B—N1B—H1NB	118.8 (14)
C6A—N1A—H1NA	117.9 (15)	C6B—N1B—H1NB	116.1 (13)
C7A—N2A—H2NA	121.1 (16)	C7B—N2B—H2NB	121.6 (14)
C7A—N2A—H3NA	118.6 (14)	C7B—N2B—H3NB	120.5 (15)
H2NA—N2A—H3NA	120 (2)	H2NB—N2B—H3NB	115 (2)
F1A—C1A—C2A	123.22 (18)	F1B—C1B—C2B	119.17 (15)
F1A—C1A—C6A	114.43 (17)	F1B—C1B—C6B	117.95 (15)
C2A—C1A—C6A	122.35 (16)	C2B—C1B—C6B	122.88 (16)
C2A—C1A—H1AA	118.8	C2B—C1B—H1BA	118.6
C6A—C1A—H1AA	118.8	C6B—C1B—H1BA	118.6
C3A—C2A—C1A	116.96 (17)	C3B—C2B—C1B	116.17 (16)
C3A—C2A—H2AA	121.5	C3B—C2B—H2BA	121.9
C1A—C2A—H2AA	121.5	C1B—C2B—H2BA	121.9
F2A—C3A—C2A	117.99 (16)	F2B—C3B—C4B	118.46 (16)
F2A—C3A—C4A	118.49 (16)	F2B—C3B—C2B	117.77 (15)
C2A—C3A—C4A	123.51 (17)	C4B—C3B—C2B	123.76 (16)
C3A—C4A—C5A	117.47 (16)	C3B—C4B—C5B	117.95 (16)
C3A—C4A—H4AA	121.3	C3B—C4B—H4BA	121.0
C5A—C4A—H4AA	121.3	C5B—C4B—H4BA	121.0
F1AX—C5A—C4A	120.96 (19)	F1BX—C5B—C6B	109.6 (6)
F1AX—C5A—C6A	117.52 (19)	F1BX—C5B—C4B	129.6 (6)
C4A—C5A—C6A	121.52 (17)	C6B—C5B—C4B	120.84 (16)
C4A—C5A—H5AA	119.2	C6B—C5B—H5BA	119.6
C6A—C5A—H5AA	119.2	C4B—C5B—H5BA	119.6
C5A—C6A—C1A	118.07 (16)	C1B—C6B—C5B	118.40 (15)
C5A—C6A—N1A	121.93 (16)	C1B—C6B—N1B	120.01 (15)
C1A—C6A—N1A	120.00 (15)	C5B—C6B—N1B	121.51 (15)
N2A—C7A—N1A	116.32 (16)	N2B—C7B—N1B	116.42 (15)
N2A—C7A—S1A	121.39 (14)	N2B—C7B—S1B	121.45 (13)
N1A—C7A—S1A	122.25 (13)	N1B—C7B—S1B	122.11 (12)
			
F1A—C1A—C2A—C3A	179.30 (18)	F1B—C1B—C2B—C3B	179.05 (14)
C6A—C1A—C2A—C3A	−1.0 (3)	C6B—C1B—C2B—C3B	−0.44 (14)
C1A—C2A—C3A—F2A	176.87 (16)	C1B—C2B—C3B—F2B	179.36 (14)
C1A—C2A—C3A—C4A	−2.1 (3)	C1B—C2B—C3B—C4B	0.66 (15)
F2A—C3A—C4A—C5A	−176.43 (15)	F2B—C3B—C4B—C5B	−179.51 (14)
C2A—C3A—C4A—C5A	2.5 (3)	C2B—C3B—C4B—C5B	−0.8 (2)
C3A—C4A—C5A—F1AX	−179.1 (2)	C3B—C4B—C5B—F1BX	−176.9 (4)
C3A—C4A—C5A—C6A	0.2 (3)	C3B—C4B—C5B—C6B	0.7 (2)
F1AX—C5A—C6A—C1A	176.2 (2)	F1B—C1B—C6B—C5B	−179.09 (14)
C4A—C5A—C6A—C1A	−3.1 (3)	C2B—C1B—C6B—C5B	0.4 (2)
F1AX—C5A—C6A—N1A	−4.8 (3)	F1B—C1B—C6B—N1B	4.3 (2)
C4A—C5A—C6A—N1A	175.88 (16)	C2B—C1B—C6B—N1B	−176.21 (13)
F1A—C1A—C6A—C5A	−176.79 (16)	F1BX—C5B—C6B—C1B	177.5 (4)
C2A—C1A—C6A—C5A	3.5 (3)	C4B—C5B—C6B—C1B	−0.6 (2)
F1A—C1A—C6A—N1A	4.3 (2)	F1BX—C5B—C6B—N1B	−5.9 (4)
C2A—C1A—C6A—N1A	−175.43 (16)	C4B—C5B—C6B—N1B	176.01 (15)
C7A—N1A—C6A—C5A	−107.0 (2)	C7B—N1B—C6B—C1B	−79.0 (2)
C7A—N1A—C6A—C1A	71.9 (2)	C7B—N1B—C6B—C5B	104.47 (19)
C6A—N1A—C7A—N2A	−169.70 (16)	C6B—N1B—C7B—N2B	174.25 (15)
C6A—N1A—C7A—S1A	12.6 (2)	C6B—N1B—C7B—S1B	−7.4 (2)

### Hydrogen-bond geometry (Å, º)

**Table:**

*D*—H···*A*	*D*—H	H···*A*	*D*···*A*	*D*—H···*A*
N1*A*—H1*NA*···S1*B*	0.794 (19)	2.586 (19)	3.3485 (15)	161.7 (19)
N2*A*—H2*NA*···S1*B*	0.81 (2)	2.77 (3)	3.499 (2)	151 (2)
N2*A*—H3*NA*···S1*B*^i^	0.85 (2)	2.65 (2)	3.504 (2)	175.2 (16)
N1*B*—H1*NB*···S1*A*^ii^	0.88 (2)	2.49 (2)	3.3273 (15)	158.9 (17)
N2*B*—H2*NB*···S1*A*^ii^	0.88 (2)	2.76 (2)	3.5179 (19)	146.4 (18)
N2*B*—H3*NB*···S1*A*^iii^	0.82 (3)	2.66 (3)	3.4592 (19)	167 (2)
C4*B*—H4*BA*···F1*B*^i^	0.95	2.50	3.094 (2)	121
C5*B*—H5*BA*···F1*B*^i^	0.95	2.52	3.111 (2)	121

Symmetry codes: (i) *x*, *y*, *z*+1; (ii) *x*−1, *y*, *z*−1; (iii) *x*, *y*, *z*−1.
